# Lipid-Based Nutrient Supplements During Pregnancy and Lactation Did Not Affect Human Milk Oligosaccharides and Bioactive Proteins in a Randomized Trial

**DOI:** 10.3945/jn.117.252981

**Published:** 2017-08-09

**Authors:** Josh M Jorgensen, Charles Arnold, Per Ashorn, Ulla Ashorn, David Chaima, Yin Bun Cheung, Jasmine CC Davis, Yue-Mei Fan, Elisha Goonatilleke, Emma Kortekangas, Chiza Kumwenda, Carlito B Lebrilla, Kenneth Maleta, Sarah M Totten, Lauren D Wu, Kathryn G Dewey

**Affiliations:** 1 Departments of Nutrition,; 2 Chemistry, and; 3 Biochemistry and Molecular Medicine, University of California, Davis, Davis, CA;; 4 Faculty of Medicine and Life Sciences, University of Tampere, Tampere, Finland;; 5 Department of Pediatrics, Tampere University Hospital, Tampere, Finland;; 6 Department of Community Health, University of Malawi College of Medicine, Blantyre, Malawi;; 7 Centre for Quantitative Medicine, Duke-National University of Singapore Graduate Medical School, Singapore, Singapore; and; 8 Department of Biostatistics, Singapore Clinical Research Institute, Singapore, Singapore

**Keywords:** human milk oligosaccharides, bioactive breast milk proteins, lipid-based nutrient supplements, multiple micronutrient supplements, lactation, postpartum

## Abstract

**Background:** Human milk oligosaccharides (HMOs) and bioactive proteins are beneficial to infant health. Recent evidence suggests that maternal nutrition may affect the amount of HMOs and proteins in breast milk; however, the effect of nutrient supplementation on HMOs and bioactive proteins has not yet been well studied.

**Objective:** We aimed to determine whether lipid-based nutrient supplements (LNSs) affect milk bioactive protein and HMO concentrations at 6 mo postpartum in women in rural Malawi. These are secondary outcomes of a previously published randomized controlled trial.

**Methods:** Women were randomly assigned to consume either an iron and folic acid capsule (IFA) daily from ≤20 wk gestation until delivery, followed by placebo daily from delivery to 6 mo postpartum, or a multiple micronutrient (MMN) capsule or LNS daily from ≤20 wk gestation to 6 mo postpartum. Breast milk concentrations of total HMOs, sialylated HMOs, fucosylated HMOs, lactoferrin, lactalbumin, lysozymes, antitrypsin, immunoglobulin A, and osteopontin were analyzed at 6 mo postpartum (*n* = 647). Between-group differences in concentrations and in proportions of women classified as having low concentrations were tested.

**Results:** HMO and bioactive protein concentrations did not differ between groups (*P* > 0.10 for all comparisons). At 6 mo postpartum, the proportions of women with low HMOs or bioactive proteins were not different between groups except for osteopontin. A lower proportion of women in the IFA group had low osteopontin compared with the LNS group after adjusting for covariates (OR: 0.5; 95% CI: 0.3, 0.9; *P* = 0.016).

**Conclusion:** The study findings do not support the hypothesis that supplementation with an LNS or MMN capsule during pregnancy and postpartum would increase HMO or bioactive milk proteins at 6 mo postpartum among Malawian women. This trial was registered at clinicaltrials.gov as NCT01239693.

## Introduction

The health benefits of human milk for infants are well recognized (1). Human milk contains numerous constituents such as proteins, fats, carbohydrates, water, electrolytes, vitamins, minerals, and other organic compounds, all of which facilitate infant growth and short- and long-term physical health. Oligosaccharides and bioactive proteins are among the organic compounds produced and transported by the mammary gland.

Human milk oligosaccharides (HMOs) are a diverse group of glycans that are highly abundant in human milk. HMOs have been shown to confer an array of benefits to infants by promoting specific bacterial colonization of the infant gastrointestinal tract. HMOs function as prebiotics for beneficial bacteria and also act as antiadhesive antimicrobials, modulate immune and intestinal epithelial cell responses, and may even benefit the mother via retrograde “leakage” into the circulation (2). Furthermore, oligosaccharides containing sialic acid and/or fucose confer additional benefits to the infant by preventing pathogenic adhesion to intestinal epithelial surfaces and possibly enhancing brain development (3).

Numerous bioactive proteins found in human milk have been shown to benefit infants by protecting against infection or improving the acquisition of nutrients from breast milk. Lactoferrin enhances iron absorption and also has bacteriostatic and bactericidal properties and modulates the infant immune system (4). Lactalbumin also enhances the absorption of iron and other minerals such as calcium and zinc. Furthermore, evidence shows that lactalbumin indirectly exhibits bactericidal and immune-stimulating activities through intraintestinal enzymatic cleavage of lactalbumin into bioactive peptides (5, 6). Lysozymes act both synergistically with lactoferrin and independently to kill Gram-negative bacteria (4). Moreover, evidence shows that the consumption of lysozymes is associated with increased expression of anti-inflammatory cytokine TGF-β and inhibits harmful Firmicutes bacteria (especially *Clostridium*) while stimulating beneficial Bacteroidetes (7). Antitrypsin inhibits the proteolytic enzyme trypsin, thereby aiding the survival of other bioactive proteins in the infant gastrointestinal tract (8). IgA is present in substantial amounts throughout lactation. Antigen-specific IgA prevents the adherence and penetration of bacterial and dietary antigens capable of provoking inflammation in the intestinal mucosa (9). In addition, osteopontin enhances immunologic development in infants (10), intestinal development in newborn rhesus monkeys (11), and neurologic and cognitive development in mouse pups (12).

We recently reported that mothers of severely stunted infants had significantly fewer sialylated HMOs in their milk than mothers of nonstunted infants within the Malawi cohort enrolled in the International Lipid-Based Nutrient Supplements (iLiNS) Project DYAD (Supplementing Maternal and Infant Diet With High-Energy, Micronutrient Fortified Lipid-Based Nutrient Supplements) trial (http://www.ilins.org) (13). We also found substantial variation in HMO content among women enrolled in the study. A limited number of studies have aimed to elucidate reasons for such variation in HMOs and bioactive proteins. Davis et al. (14) recently found seasonal differences in total HMO abundance in The Gambia, in which milk samples collected in the dry season (when food is more plentiful) had higher total HMOs than did milk samples collected during the wet season (when food is scarcer). This suggests a relation between maternal dietary intake and HMO production, although other factors could be involved. Some evidence shows that a high-protein or high-fiber diet may increase particular milk oligosaccharides in rats (15), and dietary vitamin A is also positively associated with the concentration of sialic acid in human milk (16). In addition, β-carotene supplementation and high-protein and high-calorie diets have been positively associated with particular bioactive human milk proteins (17–19), whereas “malnourishment” has been associated with low concentrations of particular milk proteins (20). Taken together, these studies suggest that maternal diet influences the production of HMOs and proteins; however, to our knowledge, no study has yet examined the effect of providing a nutritional supplement that contains both macro- and micronutrients on HMOs or bioactive milk proteins.

The iLiNS-DYAD trials in Malawi and Ghana were designed to determine whether providing lipid-based nutrient supplements (LNSs) to women during pregnancy and the first 6 mo of lactation and to children from 6 to 18 mo of age improves fetal and child growth, micronutrient status, and neurobehavioral development to a greater extent than consuming iron and folic acid (IFA) during pregnancy only or a multiple micronutrient (MMN) tablet during pregnancy and the first 6 mo of lactation. In this study, we examined the effect of LNS and MMN supplementation compared with IFA during pregnancy and placebo postpartum on HMO and bioactive milk protein concentrations, which were secondary outcomes. Given the often inadequate nutritional status among pregnant and postpartum women in Malawi and evidence from The Gambia and elsewhere that nutrition is associated with production of HMOs and proteins, we hypothesized that consumption of micro- and macronutrient supplements is associated with higher concentrations of HMOs and proteins.

## Methods

The iLiNS-DYAD-Malawi trial was a randomized controlled, outcome assessor–blinded supplementation trial of mother-child dyads in the Mangochi District of Malawi (iLiNS-DYAD-Malawi). The study is described in detail elsewhere (21). Briefly, we enrolled 1391 pregnant women who came to one of the study clinics for antenatal care who were >15 y of age and not >20 gestational weeks. Women who had chronic medical conditions, pregnancy complications at enrollment (e.g., moderate to severe edema, blood hemoglobin concentration <50 g/L, systolic blood pressure >160 mm Hg, or diastolic blood pressure >100 mm Hg), previous enrollment in the iLiNS-DYAD trial, or concurrent enrollment in another clinical trial were excluded. All 1391 women enrolled were asked to consume supplements until delivery; a subset of 869 women were asked to continue supplementation until 6 mo postpartum. Of those who were still breastfeeding at 6 mo, 647 successfully submitted a breast milk sample.

Women were randomly assigned to 1 of 3 intervention groups in blocks of 9 by selecting an opaque envelope that contained 1 of 9 letters. Each intervention group had 3 letters that corresponded to it. Women in the IFA group were instructed to consume a capsule that contained 60 mg Fe and 400 μg folic acid (**Supplemental Table 1**) each day from enrollment until delivery, and they consumed a placebo capsule from delivery to 6 mo postpartum. Women in the MMN group were instructed to consume a capsule that contained 20 mg Fe, in addition to folic acid and 16 additional micronutrients, each day from enrollment to 6 mo postpartum. The IFA, placebo, and MMN capsules were identical in appearance and were contained in bottles labeled with a letter that corresponded to the intervention. Women in the small-quantity–LNS group were instructed to consume the following each day from enrollment until 6 mo postpartum: a 20-g dose of an LNS that contained the same 18 micronutrients as the MMN capsule, 4 additional minerals, 2.6 g protein, and 10 g fat and also provided 118 kcal of energy. Fifteen supplement doses were delivered by the study staff every 14 d. The capsules were manufactured by DSM Nutritional Products South Africa (Pty) Ltd. The LNS was produced and packed by Nutriset S.A.S. The capsules and LNSs were stored in a dark environment at 20–40°C. A statistician not involved in the iLiNS-DYAD-Malawi trial (JM Peerson, University of California, Davis) maintained the intervention code, which was not broken until all laboratory and statistical analyses of the primary endpoints were performed.

Trained study staff collected sociodemographic information, including age and parity, at the enrollment visit. Information about household assets was collected and used to form a household asset *z* score (HHAZ), which is a proxy for socioeconomic status. The HHAZ includes information on the home such as the building materials used, sources of water, type of lighting used, type of cooking fuel used, and sanitary facilities. At enrollment, trained anthropometrists measured participants’ weight and height in triplicate using high-quality scales (SECA 874 flat scale; Seca GmbH & Co.) and stadiometers (Harpenden stadiometer; Holtain Limited). HIV was analyzed using a whole-blood antibody rapid test (Alere Determine HIV-1/2; Alere).

Mothers were asked to manually express the full content of one breast into a sterile plastic cup. Study staff thoroughly mixed the contents with a spoon and then transferred 10 mL to storage cryovials, which were stored at −80°C until analysis. HMOs were analyzed by a nano-LC-chip–time-of-flight MS method as described previously (22) and are reported as normalized ion counts. Lactoferrin, lactalbumin, lysozymes, antitrypsin, IgA, and osteopontin were analyzed by multiple reaction monitoring (MRM). MRM is a highly sensitive technique that has been widely used with proteins (23–26) and small molecule metabolites (27, 28) for absolute quantitation. MRM is a targeted quantitation technique and requires the molecular weight of the analyte and its fragment ions under collision-induced dissociation. In MRM, the analyte of interest is selected in the first quadrupole, fragmented in the collision cell, and 2 fragment ions (a quantifier and a qualifier) are selected and monitored in the third quadrupole for quantitation. For absolute quantitation of human milk proteins, ≥2 unique peptides from each milk protein were selected for monitoring with the MRM technique (29). Concentrations of lactoferrin, lysozymes, antitrypsin, and IgA were obtained using calibration curves of known amounts of protein standards. Osteopontin standards were not available at the time of analysis, so relative protein amounts are presented as ion counts.

We performed statistical analysis with SAS software (version 9.4; SAS Institute Inc.) according to an analysis plan that was written and published before the intervention code was opened (www.ilins.org). We based the analysis on an intention-to-treat principle. That is, results for all women enrolled were analyzed according to the group to which they were assigned regardless of any protocol violations. Data on participants who were lost to follow-up because of death, travel from the study site, or refusal to continue with the study were included in the analysis if available. Outcome variables were inspected for conformance to normal distribution and were transformed where necessary. Sialylated HMOs, lactoferrin, lysozymes, and IgA were log transformed before analyses were performed. No suitable transformation could be achieved for osteopontin, so a ranked ANCOVA approach was used to test the differences between intervention groups (30).

*P* values for differences in characteristics between women who were included and excluded from the HMO analyses were obtained from a *t* test for comparisons of means or a Fisher’s exact test for comparisons of proportions (we present these analyses only from women included and excluded from the analyses of HMOs because the women included in the HMO analyses were very similar to those included in the protein analyses). Groupwise differences in outcome variables at 6 mo postpartum were analyzed using ANCOVA for continuous outcomes and logistic regression for dichotomous outcomes. After we performed a closed testing procedure to control the type I error rate (31), a hypothesis on pairwise difference was rejected at the 5% level only if the global null hypothesis of no difference between any of the 3 groups was also rejected at the 5% level. Pairwise comparisons were performed with a Tukey-Kramer adjustment. Both unadjusted and covariate-adjusted testing of differences between intervention groups was performed. For adjusted models, covariates that have been shown to be associated with the outcome variable and were significantly associated with the outcome variable on bivariate analysis (*P* < 0.10) were included. Potential covariates included baseline maternal BMI (in kg/m^2^), HIV status, parity, maternal age, HHAZ, and season (at 6 mo postpartum). In addition to the 3-group analysis, data for the IFA and MMN groups were combined and compared with the LNS group.

The effects of potential effect modifiers were assessed with an interaction term in the ANCOVA (for continuous outcomes) or logistic regression (for bivariate outcomes). To understand the nature of the effect modification, significant interactions (*P* < 0.05) were further examined by estimating the adjusted means at the 10th and 90th percentiles of the effect modifier. The potential effect modifiers included the following variables: baseline maternal BMI, HIV status, parity, age, socioeconomic status, and season (at 6 mo postpartum).

## Results

**FIGURE 1:**
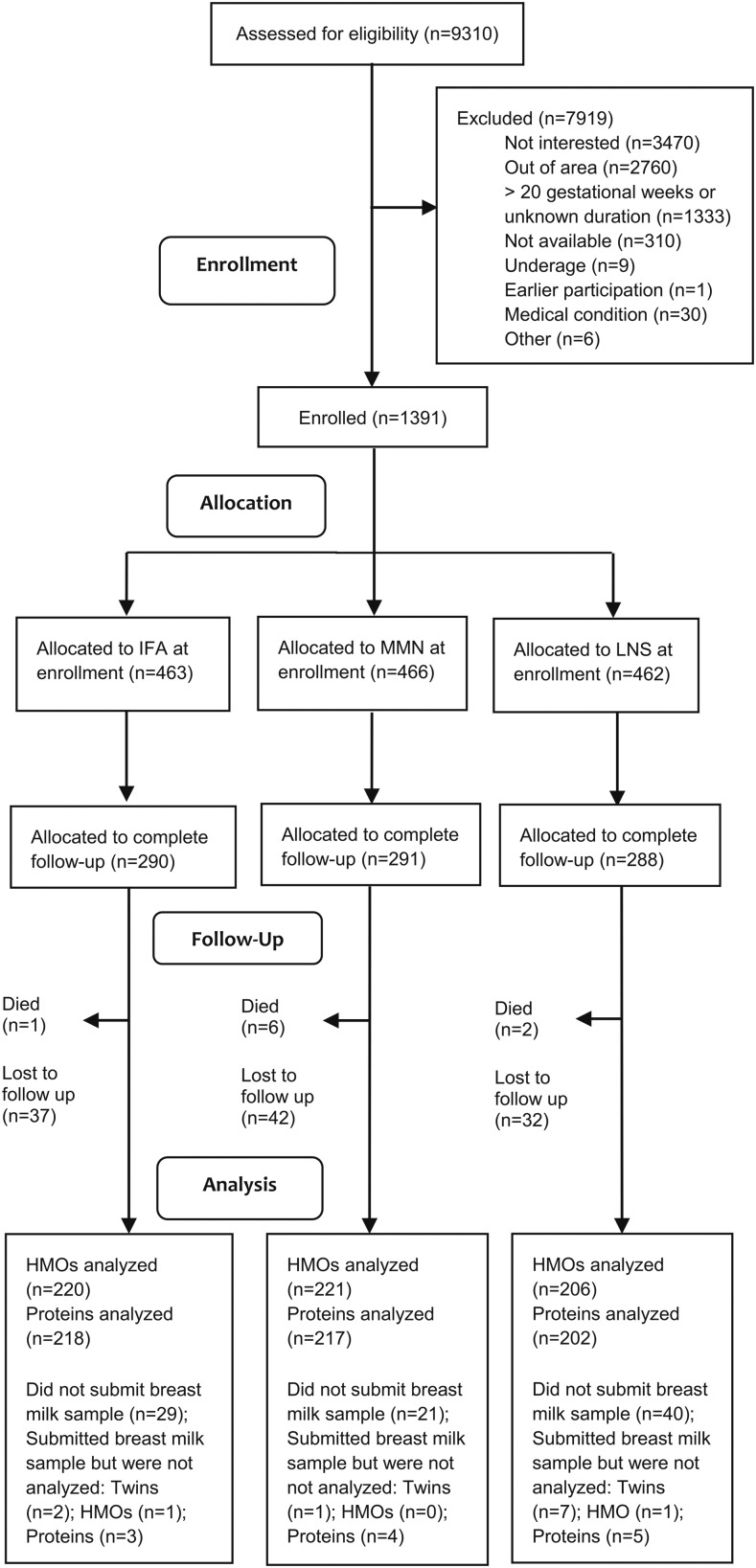
Schematic representation of recruitment, enrollment, and follow‐up of Malawian women who participated in the International Lipid-Based Nutrient Supplements Project. HMO, human milk oligosaccharide; IFA, iron and folic acid; LNS, lipid-based nutrient supplement; MMN, multiple micronutrient.

Of the 869 women selected to continue supplementation until 6 mo postpartum, 749 were still enrolled at 6 mo postpartum (**Figure 1**) and >99% were still breastfeeding. Breast milk was successfully collected from 659 women at 6 mo postpartum. After we excluded mothers of twins (*n* = 10), HMO data were available from 647 women and protein data were available from 637 women. Samples from 2 participants (1 each in the IFA and LNS groups) did not meet HMO quality control standards and samples from 12 participants (3, 4, and 5 in the IFA, MMN, and LNS groups, respectively) did not meet bioactive protein quality control standards and were excluded from analysis.

Baseline characteristics of participants included and excluded from the HMO analyses are shown in **Table 1**. Included participants had, on average, lower socioeconomic status (0.15 SD; *P* = 0.010) and lower BMI (0.5 points: *P* = 0.011) than excluded participants.

**TABLE 1:** Baseline characteristics of Malawian women included and excluded from intervention group comparisons of human milk oligosaccharides at 6 mo postpartum^1^

Characteristic	Included (*n* = 647)	Excluded (*n* = 744)	*P* value^2^
Maternal age, y	25.1 ± 6.0	24.7 ± 6.2	0.187
Proxy for socioeconomic status	−0.07 ± 1.05	0.08 ± 0.96	0.010
Primiparous women	136 (21.1)	166 (22.7)	0.473
BMI, kg/m^2^	21.9 ± 2.7	22.4 ± 3.0	0.011
Women with a low BMI (<18.5)	36 (5.6)	38 (5.2)	0.811
Women with a positive HIV test result	79 (12.3)	101 (14.9)	0.173

1 Values are means ± SDs or *n* (%).

2 *P* values are expressed for the difference in characteristics between those included and excluded from analyses, as determined by the *t* test for continuous variables and by Fisher’s exact test for comparisons of proportions.

At 6 mo postpartum, there were no significant differences in mean concentrations of total HMOs, sialylated or fucosylated HMOs, lactoferrin, lactalbumin, lysozymes, antitrypsin, IgA, or osteopontin between the 3 intervention groups in either the unadjusted or adjusted models (each *P* > 0.10) (**Table 2**). There were also no differences between groups when the IFA and MMN groups were combined and compared with the LNS group in either unadjusted or adjusted models (each *P* > 0.10, data not shown).

**TABLE 2:** HMOs and bioactive proteins at 6 mo postpartum among groups of Malawian women who consumed different formulations of micronutrient supplements during pregnancy and until 6 mo postpartum^1^

					Comparison^3^					
					IFA and MMN		IFA and LNS		MMN and LNS	
Variable	IFA	MMN	LNS	*P* value^2^	MD	*P* value	MD	*P* value	MD	*P* value
Total HMOs^4^	0.67 ± 0.01 (220)	0.69 ± 0.01 (221)	0.70 ± 0.01 (206)	0.353	−0.02 (−0.07, 0.02)	0.460	−0.03 (−0.07, 0.02)	0.396	−0.003 (−0.05, 0.04)	0.991
Sialylated HMOs^5^	0.079 ± 0.028 (220)	0.083 ± 0.032 (221)	0.083 ± 0.032 (206)	0.514	−0.04 (−0.01, 0.00)	0.700	−0.004 (−0.01, 0.00)	0.504	−0.001 (−0.01, 0.01)	0.943
Fucosylated HMOs	0.41 ± 0.01 (220)	0.43 ± 0.01 (221)	0.41 ± 0.01 (206)	0.317	−0.02 (−0.05, 0.01)	0.313	−0.01 (−0.04, 0.03)	0.936	0.02 (−0.02, 0.05)	0.524
Lactoferrin	1.05 ± 0.47 (218)	1.01 ± 0.37 (216)	1.03 ± 0.44 (202)	0.990	0.04 (−0.06, 0.14)	0.989	0.02 (−0.08, 0.12)	0.996	−0.02 (−0.12, 0.08)	0.998
Lactalbumin	1.34 ± 0.02 (218)	1.34 ± 0.01 (217)	1.35 ± 0.02 (202)	0.914	−0.005 (−0.06, 0.05)	0.974	−0.01 (−0.06, 0.04)	0.906	−0.005 (−0.06, 0.05)	0.976
Lysozymes	0.061 ± 0.049 (218)	0.058 ± 0.042 (217)	0.057 ± 0.041 (202)	0.610	0.03 (−0.01, 0.01)	0.797	0.004 (−0.01, 0.01)	0.592	0.001 (−0.01, 0.01)	0.936
Antitrypsin	0.037 ± 0.001 (218)	0.035 ± 0.001 (217)	0.036 ± 0.001 (202)	0.546	0.001 (−0.00, 0.00)	0.521	0.001 (−0.00, 0.00)	0.781	−0.001 (−0.00, 0.00)	0.916
IgA	0.33 ± 0.31 (218)	0.33 ± 0.12 (217)	0.35 ± 0.16 (202)	0.344	0.04 (−0.01, 0.09)	0.332	0.02 (−0.03, 0.07)	0.921	−0.02 (−0.07, 0.03)	0.574
Osteopontin	73,953 ± 46,480 (218)	66,844 ± 44,317 (217)	66,858 ± 46,114 (202)	0.272	7109	0.319	7096	0.375	−13	0.997

1 Values are means ± SDs (*n*), means ± SEMs (*n*), or MDs (95% CIs) unless otherwise indicated. Normalized ion counts are given for total, sialylated, and fucosylated HMOs and ion counts are given for osteopontin. All other values are given in grams per liter. HMO, human milk oligosaccharide; IFA, iron and folic acid; LNS, lipid-based nutrient supplement; MD, mean difference; MMN, multiple micronutrient.

2 Unadjusted global *P* values are presented for differences between 3 intervention groups as determined by ANOVA. After adjusting for covariates, there continued to be no differences between intervention groups for any of the outcomes. Sialylated HMOs, lactoferrin, lysozymes, and IgA were log transformed before analyses were performed. *P* values for the difference in osteopontin were determined by using ranks.

3 Values are expressed as MDs (95% CIs) of untransformed data. 95% CIs for pairwise comparisons of osteopontin could not be reliably estimated because of the highly skewed distribution. *P* values are presented for unadjusted pairwise comparisons as determined by ANOVA. Analyses were performed on untransformed variables, except for sialylated HMOs, lactoferrin, lysozymes, and IgA, which were log transformed before analysis. Analyses for osteopontin were performed using ranked ANCOVA.

4 Values are expressed as means ± SEs (*n*) for the following untransformed variables: total HMOs, fucosylated HMOs, lactalbumin, and antitrypsin. SEs are presented because these variables did not need transformation.

5 Values are expressed as means ± SD (*n*) for the following transformed variables: sialylated HMOs, lactoferrin, lysozymes, IgA, and osteopontin. SDs are presented because these variables, other than osteopontin, were log transformed, rendering back transformation of SEs difficult.

There were no significant differences in the proportion of women with low concentrations of total HMOs, sialylated HMOs, fucosylated HMOs, lactoferrin, lactalbumin, lysozymes, antitrypsin, or IgA between the 3 intervention groups in either adjusted or unadjusted models (each *P* > 0.10) (**Table 3**) or when the combined IFA and MMN group was compared with the LNS group (data not shown). There was a trend toward reduced odds of women in the IFA group having low osteopontin compared with the LNS group in unadjusted models (*P* = 0.095 for the overall model; *P* = 0.083 for IFA compared with LNS). The difference became significant after adjusting for covariates (*P* = 0.021 for the overall model; OR: 0.5; 95% CI: 0.3, 0.9; *P* = 0.016 for IFA compared with LNS). When the combined IFA and MMN group was compared with the LNS group, the proportion of women with low osteopontin did not differ in unadjusted models (*P* = 0.106); after adjusting for covariates, women in the IFA and MMN group had reduced odds of having low osteopontin (OR: 0.6; 95% CI: 0.4–0.96; *P* = 0.030; data not shown).

**TABLE 3:** Proportion of participants with HMOs and bioactive proteins below specified cutoffs among Malawian women who consumed different formulations of micronutrients during pregnancy and until 6 mo postpartum^1^

					Comparison					
					IFA and MMN		IFA and LNS		MMN and LNS	
Variable	IFA	MMN	LNS	*P* value^2^	OR (95% CI)	*P* value	OR (95% CI)	*P* value	OR (95% CI)	*P* value
Total HMOs <0.55	57 (25.9)	54 (24.2)	49 (23.8)	0.872	1.1 (0.7, 1.8)	0.932	1.1 (0.7, 1.9)	0.868	1.0 (0.6, 1.8)	0.987
Sialylated HMOs <0.060	55 (25.0)	58 (26.2)	50 (24.3)	0.893	0.9 (0.6, 1.6)	0.952	1.0 (0.6, 1.8)	0.983	1.1 (0.7, 1.9)	0.886
Fucosylated HMOs <0.33	57 (25.9)	53 (24.0)	51 (24.8)	0.895	1.1 (0.7, 1.9)	0.887	1.1 (0.6, 1.8)	0.960	1.0 (0.6, 1.6)	0.981
Lactoferrin <0.74	53 (24.3)	54 (24.9)	51 (25.3)	0.975	1.0 (0.6, 1.6)	0.989	1.0 (0.6, 1.6)	0.973	1.0 (0.6, 1.7)	0.996
Lactalbumin <1.2	55 (25.2)	56 (25.8)	49 (24.3)	0.934	1.0 (0.6, 1.6)	0.990	1.1 (0.6, 1.8)	0.971	1.1 (0.6, 1.9)	0.929
Lysozyme <0.030	48 (22.0)	56 (25.8)	56 (27.7)	0.389	0.8 (0.5, 1.4)	0.624	0.7 (0.4, 1.3)	0.368	0.9 (0.5, 1.5)	0.898
Antitrypsin <0.026	49 (22.5)	51 (23.5)	60 (29.7)	0.188	0.9 (0.6, 1.6)	0.965	0.7 (0.4, 1.2)	0.212	0.7 (0.4, 1.2)	0.324
IgA <0.25	53 (24.3)	53 (24.4)	51 (25.3)	0.971	1.0 (0.6, 1.7)	>0.999	1.0 (0.6, 1.6)	0.973	1.0 (0.6, 1.6)	0.979
Osteopontin <18,200	44 (20.2)	57 (26.3)	59 (29.2)	0.095	0.7 (0.4, 1.2)	0.292	0.6 (0.4, 1.1)	0.083^3^	0.9 (0.5, 1.4)	0.780

1 Values are *n* (%) unless otherwise indicated. Normalized ion counts are given for total, sialylated, and fucosylated HMOs and ion counts are given for osteopontin. All other values are given in grams per liter. HMO, human milk oligosaccharide; IFA, iron and folic acid; LNS, lipid-based nutrient supplement; MMN, multiple micronutrient.

2 Unadjusted global *P* values are presented for differences between 3 intervention groups as determined by logistic regression. After adjusting for covariates, no comparisons became statistically significant, other than osteopontin.

3 After adjusting for covariates, this difference became significant (*P* = 0.021 for the overall model; *P* = 0.016 for IFA compared with LNS).

Interactions of treatment group with either maternal HIV status or the season during which the breast milk sample was collected were not significant for any of the outcomes (each *P* > 0.05). The association between the intervention group and mean IgA was modified by maternal BMI (*P* = 0.008) (**Table 4**). Specifically, the MMN group had higher IgA than the IFA group among women with a low BMI, but there were no group differences among those with a higher BMI. For low fucosylated HMOs, there was a significant interaction between the intervention group and HHAZ (*P* = 0.048), but the analyses at different concentrations of HHAZ did not show consistent results. The proportion of women with low antitrypsin was modified by parity (*P* = 0.012) and maternal age (*P* = 0.046). There was a greater proportion of women with low antitrypsin in the LNS group than the IFA group among primiparous women and young women, but there were no between-group differences for multiparous or older women.

**TABLE 4:** Significant interactions with the effect of intervention on mean breast milk IgA and proportions with low fucosylated HMOs or antitrypsin at 6 mo postpartum among Malawian women who consumed different formulations of micronutrient supplements during pregnancy and until 6 mo postpartum, by baseline level of the effect modifiers^1^

Outcome	Level of effect modifier	IFA	MMN	LNS	*P*-interaction^2^	*P* value^3^
IgA	BMI				0.008	
	10th percentile	0.33 (0.30, 0.36)^a^	0.27 (0.25, 0.30)^b^	0.29 (0.26, 0.32)^a,b^		0.003
	90th percentile	0.30 (0.27, 0.33)	0.33 (0.30, 0.37)	0.34 (0.31, 0.37)		0.121
Low fucosylated HMO	HHAZ				0.048	
	10th percentile	0.26 (0.20, 0.34)	0.17 (0.12, 0.24)	0.23 (0.17, 0.31)		0.174
	90th percentile	0.26 (0.17, 0.37)	0.37 (0.27, 0.48)	0.27 (0.17, 0.38)		0.270
Low antitrypsin	Parity				0.012	
	Primiparous	0.10 (0.06, 0.17)^a^	0.16 (0.10, 0.24)^a,b^	0.29 (0.20, 0.41)^b^		0.003
	Multiparous	0.44 (0.31, 0.59)	0.32 (0.21, 0.45)	0.29 (0.19, 0.42)		0.234
	Maternal age				0.046	
	10th percentile	0.15 (0.09, 0.26)^a^	0.24 (0.15, 0.35)^a,b^	0.35 (0.25, 0.48)^b^		0.015
	90th percentile	0.28 (0.18, 0.41)	0.20 (0.12, 0.32)	0.22 (0.13, 0.34)		0.459

1 Values are estimated means (95% CIs) at the 10th and 90th percentiles of the effect modifier for continuous outcome variables as determined by ANCOVA after adjusting for covariates unless otherwise indicate. The estimated probability of low outcomes (95% CIs) at the 10th and 90th percentiles of the effect modifier for binary outcomes was determined by logistic regression after adjusting for covariates. IgA was log transformed before analyses were performed. IgA was adjusted for HHAZ, maternal HIV status, maternal BMI (in kg/m^2^), and season. Low fucosylated HMO was adjusted for HHAZ and season. Low antitrypsin was adjusted for maternal age, parity, and season. Values with different superscript letters were significantly different (*P* < 0.05) in pairwise comparisons. HHAZ, household asset *z* score; HMO, human milk oligosaccharide; IFA, iron and folic acid; LNS, lipid-based nutrient supplement; MMN, multiple micronutrient.

2 *P* values are presented for the interaction of group × effect modifier on the outcome variable.

3 Global *P* values are presented for the 3-group comparison.

## Discussion

To our knowledge, this is the first study to examine the effect of MMN supplements or LNSs on breast milk oligosaccharides (total, fucosylated, and sialylated HMOs) and bioactive proteins (lactoferrin, lactalbumin, lysozymes, antitrypsin, IgA, and osteopontin). We found that consumption of MMNs as either an MMN capsule or within an LNS during pregnancy and for 6 mo postpartum had no impact on mean breast milk concentrations of oligosaccharides or bioactive proteins at 6 mo postpartum. However, women in the control group (IFA consumed during pregnancy, followed by a placebo capsule from delivery to 6 mo postpartum) were less likely to have low milk osteopontin concentrations at 6 mo postpartum.

To date, very few studies have examined the effect of consumption of particular nutrients on HMOs. Rat dams fed either a high-protein or high-fiber diet, compared with a control diet, had higher concentrations of 2 particular milk oligosaccharides (15): hexose plus 1 N-Acetylglucosamine, which is neither sialylated nor fucosylated, yet appears to be important for the development of the intestinal microbiota and is strongly related to particular microbes found in breast-fed infants; and 2 hexose plus sialic acid, a sialylated HMO used by *Bacteroides infantis* that may help prevent the adherence of pathogens to the epithelial surface. However, in this study, the relative increase in protein intake among women in the LNS group than in the IFA or MMN groups was much lower than the relative increase in the protein and fiber intake of the group of rats that consumed the high-protein or high-fiber rat chow and was likely not large enough to affect HMO content. A separate cross-sectional study of Chinese women showed a positive association between dietary vitamin A intake reported via a 72-h food recall and sialic acid in breast milk, and a higher reported vitamin A intake was reported among women with high concentrations of sialylated HMOs (16). Our intervention trial did not show differences in sialylated HMOs among women who were supplemented with 800 μg retinol equivalents/d (in LNSs or MMNs) compared with IFA.

There is a bit more evidence regarding associations of dietary components with specific human milk proteins. Pregnant and lactating mice supplemented with 50 mg of β-carotene/kg body weight had increased milk secretion of IgA compared with mice fed a control diet (17). However, none of the supplements consumed by women in our study contained β-carotene. In addition, the breast milk IgA concentration was higher in Guatemalan women randomly assigned to receive a high-energy (500 kcal/d) or a low-energy (140 kcal/d) supplement during lactation (18). In other studies, milk lactoferrin and α-lactalbumin concentrations tended to be higher when women (*n* = 3) received a high-protein diet (20% of kcal from protein) than a low-protein diet (8% of kcal from protein) (19), and milk lysozyme and IgA concentrations were lower among women considered to be malnourished (*n* = 10; BMI, serum total protein, and albumin below established cutoffs) than in “well-nourished” women (*n* = 50) (20). The fact that we found no differences between intervention groups in these bioactive proteins may be attributable to the relatively low energy and protein content of the LNS compared with supplements used in other studies.

The lower odds of low osteopontin among women in the IFA group than in the LNS group may be a spurious association; however, it could be related to the higher dose of iron during pregnancy provided to women in the IFA group (60 mg/d) than to those in the LNS (or MMN) group (20 mg/d). In Wistar rats, serum osteopontin was increased among those fed a 5% iron lactate diet for 4 wk postpartum compared with those fed a control diet (32). Although the authors did not propose a mechanism by which iron increased osteopontin, they reported higher oxidative stress within the iron group, and osteopontin was shown in a separate study to be elevated during oxidative stress (33). Milk osteopontin was not measured in the study above; however, plasma and breast milk osteopontin were found to be significantly correlated among 20 postpartum women (34). The influence of iron consumed during pregnancy or postpartum on breast milk osteopontin concentration warrants further investigation, because osteopontin is shown to be associated with an improved cytokine profile and decreased infections in infants (35) and improved neurologic and cognitive development in mouse pups (12).

We found a few statistically significant interactions for some of the milk outcomes between certain maternal characteristics and the intervention group. Among women with a low BMI, the mean breast milk IgA concentration was higher among those who consumed IFA than MMN or LNS, but no such differences were observed among women with a higher BMI. Atlhough this may be a spurious finding, it is possible that women with a low BMI may not consume adequate iron in their diet compared with women with higher BMI (36), and the higher dose of iron in the IFA group than in the MMN and LNS groups may have been sufficient to maintain higher amounts of breast milk IgA. We could find no studies on the association between maternal iron status and breast milk IgA, although IgA has been shown to be significantly lower in the colostrum of anemic women than in nonanemic women (37). Among young primiparous women, those in the IFA group had lower odds of low antitrypsin than did those in the LNS group, whereas there were no group differences among older or multiparous women. It is plausible that the higher dose of iron consumed during pregnancy by young primiparous women in the IFA group positively influenced the production of antitrypsin, although we could find no evidence in the literature that iron status affects milk antitrypsin.

The strengths of this study include the large sample size, frequent follow-up with participants throughout the intervention period, and well-trained study staff. With regard to limitations, those included in this analysis were of lower socioeconomic status and had lower BMI values than those excluded from the study, which may limit the generalizability of the findings to the greater population. Although the differences between included and excluded participants were significant, they were quite small. Another potential limitation is that multiple outcomes were examined, so the significant finding for low osteopontin could be attributable to chance.

In conclusion, this study does not support the hypothesis that providing LNSs or MMNs during pregnancy and ≤6 mo postpartum impacts mean concentrations of HMOs or particular bioactive proteins among lactating women at 6 mo postpartum in rural Malawi. Other factors (including genetics) may be more influential than diet with regard to these human milk constituents. Further research is needed to elucidate which maternal factors, if any, can be modified to enhance the concentration of beneficial HMOs and proteins in breast milk.
